# Design and Experimental Validation of a USBL Underwater Acoustic Positioning System

**DOI:** 10.3390/s16091491

**Published:** 2016-09-14

**Authors:** Joel Reis, Marco Morgado, Pedro Batista, Paulo Oliveira, Carlos Silvestre

**Affiliations:** 1 Department of Electrical and Computer Engineering, Faculty of Science and Technology, University of Macau, Macao, China; csilvestre@umac.mo; 2 Institute for Systems and Robotics, Instituto Superior Técnico, Universidade de Lisboa, Lisboa 1049-001, Portugal; marco.m.morgado@gmail.com (M.M.); pbatista@isr.tecnico.ulisboa.pt (P.B.); pjcro@isr.ist.utl.pt (P.O.); 3 LAETA—Associated Laboratory for Energy, Transports and Aeronautics, IDMEC—Institute of Mechanical Engineering, Instituto Superior Técnico, Universidade de Lisboa, Lisboa 1049-001, Portugal

**Keywords:** underwater Ultra-Short BaseLine (USBL) system, acoustic localization, spread spectrum modulation

## Abstract

This paper presents the steps for developing a low-cost POrtableNavigation Tool for Underwater Scenarios (PONTUS) to be used as a localization device for subsea targets. PONTUS consists of an integrated ultra-short baseline acoustic positioning system aided by an inertial navigation system. Built on a practical design, it can be mounted on an underwater robotic vehicle or be operated by a scuba diver. It also features a graphical user interface that provides information on the tracking of the designated target, in addition to some details on the physical properties inside PONTUS. A full disclosure of the architecture of the tool is first presented, followed by thorough technical descriptions of the hardware components ensemble and the software development process. A series of experiments was carried out to validate the developed prototype, and the results are presented herein, which allow assessing its overall performance.

## 1. Introduction

Upon the advent of consumer-oriented marketing of navigation systems, e.g., the Global Positioning System (GPS), the topic of real-time localization reached important areas of engineering, such as cost-efficient trajectory planning and tracking of maritime, air or land vehicles. The endeavors to replicate the same tasks in underwater scenarios are also noteworthy because the properties of the medium preclude the use of GPS-based localization systems, i.e., operating scenarios like oceans, seas, rivers, estuaries, etc. present high attenuation to electromagnetic signals. Positioning aided by Radio-Frequency (RF) communications is thus impractical and a non-viable option. The fact that water owns that intrinsic property, often designated as opacity, makes acoustic signal propagation a viable alternative method. With respect to underwater telemetry, the progress in the field has been widely reviewed in [1], while in [2], the main techniques and challenges posed by the underwater source localization problem are reported.

One of the key forerunners of underwater research expansion was deep sea exploration, driven by oil and gas drilling ventures taking place predominantly in remote (offshore) zones. Examining the risks undertaken, Remotely-Operated Vehicles (ROVs) have become a liability; see [3,4,5]. ROVs are hefty machines mainly intended to perform bathymetry tasks, inspection and robotic manipulation, which conduce to a better understanding of the sea-floor bathymetry and how it may impact on marine life in the vicinity. Due to operational, safety and financial reasons, accurately perceiving the position of ROVs with respect to the surface vessels is a major requirement, which can be accomplished by resorting to Ultra-Short BaseLine (USBL) acoustic systems. In light of the above, several acoustic positioning solutions have been developed in the past. To name just a few, see, for instance, the USBL positioning system called POSIDONIA [6] and the high-performance systems Sonardyne© Ranger 2 [7] and iXBlue© GAPS [8]. Nonetheless, Easytrak USBL systems from Applied® Acoustics [9] and R-Series USBL [10] from EvoLogics® also provide efficient solutions for a wide range of subsea applications, albeit more compact than the previous counterparts.

This sustained ambition of exploring in greater detail underwater environments has led to an increase in the production of autonomous underwater vehicles (AUVs). A detailed review of the navigation and localization of AUVs is presented in [11], where the authors overview both a myriad of sensor-based methods to acquire important measurements, as well as a set of mathematical tools aiming at optimizing the robustness of those same measurements.

This notwithstanding, there has persisted a conscious need for diver localization strategies that will allow a more accurate human intervention in underwater mission scenarios [12,13]. In turn, the past MAST/AMproject, whose goal was to endow the scientific community with new moderate cost robotic tools able to track multiple targets, resulted in the construction of the first underwater prototype [14,15] at the Institute for Systems and Robotics (ISR), Lisbon. Building upon these developments, a new enhanced concept, POrtable Navigation Tool for Underwater Scenarios (PONTUS), is presented in this paper along with the details of its development process. The prototype consists of an Inertial Navigation System (INS)-aided USBL portable underwater robotic tool designed to be operated by a scuba diver or to be mounted on a AUV, featuring a graphical user interface (GUI) for visually-aided diver navigation.

Despite the advantages (and inherent disadvantages) offered by other acoustic positioning systems, such as the Long BaseLine (LBL) and short baseline techniques, our choice for the USBL technique was mainly motivated by reasons related to the design of a low cost and fast deployable localization tool for underwater environments. For instance, the LBL technique is known for being expensive and requires an a priori calibration stage any time the operation area is changed. Moreover, from a scuba diver’s perspective, we aimed at developing a fast-deployable and highly-maneuverable prototype useful in target tracking scenarios. For example, suppose a scuba diver wants to keep tracking the position of the mother-ship, or vice versa [13]. In this case, resorting only to our prototype allows for a quick and convenient solution.

Hence, in light of the main objective underlying the development of PONTUS and as opposed to the aforementioned commercial solutions, we aim to provide the scientific community with a versatile and high-performance low-cost tool for underwater tracking of moving targets, presenting a highly-configurable array, in addition to enabling access to the physical variables of the system, which, in the future, can be used in the design of novel tightly-coupled algorithms for localization and navigation. Furthermore, the implemented acoustic communications between the transponders and the USBL array (or arrays), which relies on Direct Sequence Spread Spectrum (DSSS) modulated signals, allows for operations consisting of multiple simultaneous users.

**Figure 1:**
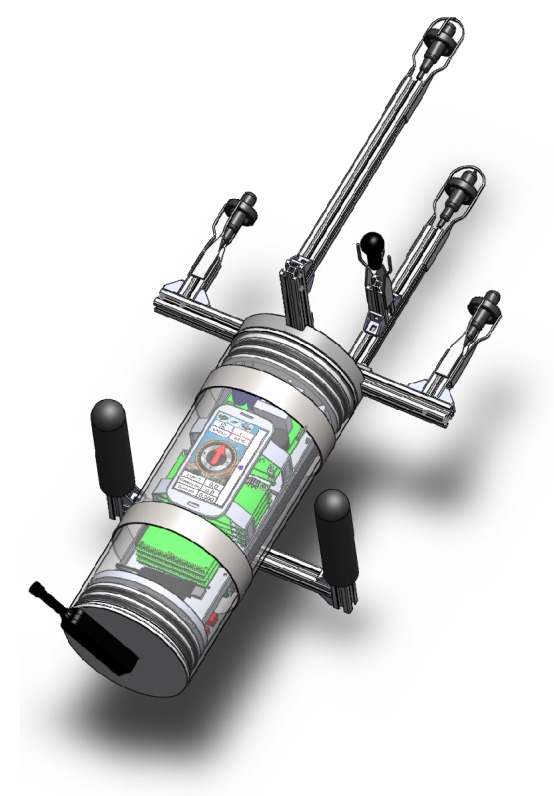
POrtable Navigation Tool for Underwater Scenarios (PONTUS) concept design in SolidWorks©.

The total cost of the construction and development of the prototype presented in this paper was approximately $6,000, excluding labor. The labor costs amounted to one year of a master student and one year of a PhD student. The full concept was modeled in SolidWorks© (Waltham, MA, USA) and can be seen in Figure 1.

This paper is organized as follows: Section 2 outlines the structure of PONTUS and the structural building materials. Section 3 details the mathematical background that provides the basis for handling acoustic signals. Section 4 presents the electric and electronic characteristics of the system and the hardware components that support it. Section 5 starts by depicting the data networks of PONTUS and then clarifies the development steps in implementing the GUI. Experimental results to assess the performance of the prototype can be found in Section 6. Conclusions along with further discussions are offered in Section 7.

### Notation

In this work, source stands for a target equipped with an acoustic transmitter. Array denotes a rigid mechanical structure comprising a set of acoustic receivers and transmitters. Baseline is the term that indicates the distance between a pair of acoustic receivers. In order to avoid ambiguity, an acoustic receiver will be henceforward addressed as a hydrophone, whereas receiver denotes the overall acoustic system in one of its operation modes. Acoustic projector is a device that converts an electrical signal into an acoustic wave, while transmitter designates the acoustic system in another operation mode. Hence, in summary, PONTUS works both as a receiver and a transmitter, housing a USBL array that comprises a set of hydrophones and one acoustic projector. For a brief mathematical context, the special orthogonal group is denoted by $SO\left(3\right):=\{X\in R^{3\times 3}:XX^{T}=X^{T}X=I\wedge \det\left(X\right)=\;1\}$, and the superscript ${\left(\cdot \right)}^{T}$ denotes the transpose operator.

## 2. Architecture of PONTUS

Conceptually, our goal is to design an underwater portable tool whose dimensions and interfaces must allow for human handling and installation onboard AUVs. This section introduces the underwater sensor and proceeds to describe the building process of the outer shell of PONTUS and how both are combined.

### 2.1. USBL Array

**Figure 2:**
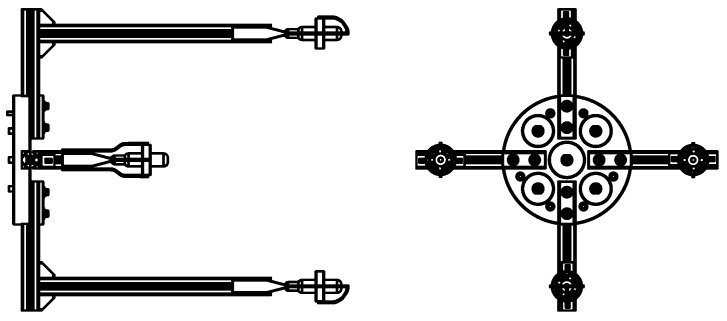
Geometry of the USBL in SolidWorks©: Top/side view on the left; front view on the right.

Determining non-ambiguous 3D source positions demands an array comprising at least four hydrophones assembled in a non-planar configuration. The geometry of this array depends mainly on the signal wavelength. Furthermore, the hydrodynamics need to be taken into account (see [6]), especially under quick maneuvers. Towards our goal, the number of hydrophones was chosen so as to obtain a simple yet effective solution: a quartet of hydrophones was selected and arranged in a semi-spherical configuration, as depicted in Figure 2. In [16,17], a different kind of arrangement and a configuration based on a square-pyramid consisting of five acoustic receivers were studied, respectively.

The specifications of the acoustic units implemented in PONTUS are in line with the need to have a light and compact tool, allowing the hydrophones to be a few centimeters apart in a highly configurable rigid structure. The maximum horizontal/vertical distance between a pair of hydrophones is 30 cm, whereas the shortest is 15 cm.

**Figure 3:**
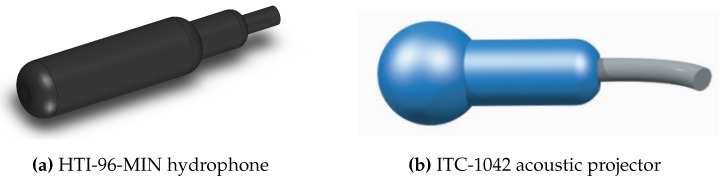
USBL’s acoustic elements.

The array includes four High Tec, Inc HTI-96-MIN hydrophones (Long Beach, MS, USA) (Figure 3a) and one ITC-1042 (Channel Technologies Group, Santa Barbara, CA, USA) Spherical Omnidirectional emitter (Figure 3b) located at the origin of the USBL sensor’s body frame. The hydrophones offer a flat frequency response from 2 Hz up to 30 kHz while the acoustic projector (emitter) presents a resonant frequency at 79 kHz. These hydrophones and the acoustic projector were chosen for their quality (in terms of omni-directionality and sensitivity) to price ratio, in addition to their reduced dimensions. In terms of materials, the array is built with Bosch-Rexroth© (Lohr am Main, Germany) aluminum rods and connections. The resulting structure is coupled to a specially-designed circular device machined in Delrin© (Wilmington, DE, USA) highly-resistant polymer plastic.

### 2.2. Outer Shell

**Figure 4:**
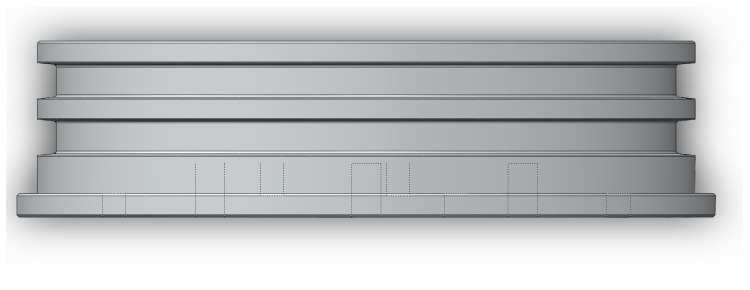
Horizontal lid profile in SolidWorks©. Dashed lines represent the holes.

The cylindrical architecture, hereafter tube, is a popular shape since its circular cross-section leads to an optimal distribution of pressure load along that section, and for that reason, it was the one selected for housing the electronics and power supplies. Furthermore, in order for the GUI to be accessed by the diver during manned operations, the tube had to be transparent, while weight is also an important factor influencing the handleability of the prototype. Therefore, an acrylic glass (much lighter than aluminum) cylinder was selected, with 8 mm in thickness, 142 mm in internal diameter and a total length of 380 mm. Each opening of the tube is enclosed with an aluminum lid. For sealing purposes, both lids have two parallel grooves around their circular surface, designed in a way that two circular cross-section O-rings can be seated in their perimeter, as shown in Figure 4.

The fore lid (in contact with the array) houses four Impulse© IE55 (Teledyne Oil & Gas, Daytona Beach, FL, USA) connectors, one for each hydrophone. Externally, the array is fixed onto this same lid. Internally, the fore lid is screwed to an aluminum tray used as a platform to attach all of the hardware. In turn, the aft lid has two holes: one is for a Low Profile 9 Pin SubConn© (The MacArtney Underwater Technology Group, Esbjerg, Denmark) connector providing outbound connections, whereas the second hole is used to pressurize and depressurize the tube. Indeed, depressurization is what induces an inwards force acting over both lids, therefore hermetically sealing PONTUS. Prior to any underwater mission, the pressure value inside the tube is brought down to circa 0.5 bar, a value that must be monitored via the GUI. Based on pressure chamber tests, the sealed tube has a maximum depth capability of 60 m (roughly seven bars).

The handling is ensured by two rubber-covered aluminum handles linked by two stainless steel rings surrounding the acrylic glass tube, as observed in Figure 1. In terms of mass, the center of gravity is displaced towards the fore lid, and the fully equipped tool weighs around 60 N in the air. However, given its volume, and the water density, the total equivalent water displacement is around 72 N, resulting in a positive buoyancy.

## 3. Algorithms within PONTUS

This section starts by introducing the different operation modes of PONTUS. Later, it describes in detail the procedures on signal acquisition, generation and processing, as well as on data processing, concluding with the presentation of the techniques employed to obtain the bearing and range measurements from raw data.

### 3.1. Modes of Operation

The versatile design of PONTUS allows for the following three modes of operation:

**(O1)**  Interrogation scheme: As the receiver, PONTUS interrogates a target (or targets) equipped with a transponder, waits for the reply and determines the range between the target and itself based on the Round-Trip-Travel time (RTT) of the acoustic signals. This notwithstanding, there must be a known response delay time induced in the target’s reply, which, upon reception of the signals, is subtracted by PONTUS from its own RTT.
**(O2)**  Passive reception: This technique can be implemented when the available acoustic transmitters do not offer interrogation-based solutions, i.e., do not allow for explicit range measurements. In order to overcome this problem (see the previous work by the authors [18]), a new localization filtering technique applied to underwater scenarios is proposed that determines the distances based on direction and Doppler readings, exclusively resorting to a single sensor.
**(O3)**  Synchronized reception: In the absence of interrogation schemes, if PONTUS and the target (or targets) are connected to GPS antennas, a synchronization between both devices is made possible by resorting to the pulse-per-second signal provided by a GPS receiver module. To avoid ambiguities, the travel time cannot exceed 1 s; hence, this configuration is only suitable for operations where slant-ranges are below 1500 m.

For all of the above methods, the determination of the corresponding Direction Of Arrival (DOA) is calculated from the USBL array readings, specifically the Time Difference Of Arrival (TDOA) measurements.

### 3.2. Acoustic Signals

From the many modulation techniques available in the literature, we selected the DSSS modulation of acoustic signals for an improved performance. DSSS modulation presents high (ambient or jamming) noise immunity, thus yielding a better Signal to Noise ratio (SNR) in the aftermath of matched filters; see, e.g., [19,20]. It also provides better multipath rejection, while offering frequency and time diversity [21], fundamental to track multiple targets simultaneously. For the particular case of PONTUS, the acoustic signals involved in the operations consist of Binary Phase Shift Keying (B-PSK)-modulated DSSS signals based on a 25-kHz sinusoidal carrier wave. A 127-chip Gold Code modulates the carrier wave with a chip rate equal to one full period of the carrier wave, spanning 5.08 ms in time.

### 3.3. Signal Processing

While listening to the underwater channel, the output of a single hydrophone is processed in real time by a matched filter-based detection algorithm. When the expected signal occurs, all data related to the remaining hydrophones are included in this algorithm. A First In First Out (FIFO) buffer is implemented for each hydrophone. In the following, the algorithm will be detailed for a single target. In the case of multiple targets, we just need to run one detection algorithm for each target.

Let *L* be the number of samples that corresponds to the length of the expected acoustic signal. Each FIFO buffer is divided into three blocks, and for data processing convenience, each block is of length *L*. Let sampling cycle denote the time it takes to fill an entire block of a FIFO buffer, given by $L/f_{s}$, where $f_{s}$ is the sampling frequency. At each sampling cycle, the first in blocks are filled with new samples, whereas the remaining are filled with samples shifted from previous sampling cycles. The content of one first in block is fed to a matched filter, whose output indicates whether or not part of the expected signal arrived during that sampling cycle.

Hence, let x[n] be the sequence that represents the discrete-time input signal, and let X[k] be the corresponding Discrete Fourier Transform (DFT). The DFT of the expected signal sequence h[n], which corresponds to the impulse response of the matched filter, denoted by H[k], is stored in memory. Let y[n] be the discrete-time output of the matched filter, with y[n]=h[n]∗x[n], where ∗ denotes the convolution operator. Convolutions are computationally heavy and have a complexity of $O(L^{2})$. However, the convolution theorem duality of the DFT states that a convolution in time can be represented by a multiplication in the frequency domain, and vice versa. It follows,

(1) Y[k]=H[k]X[k].

After the point-wise multiplication in Equation (1), the inverse DFT of the sequence Y[k] is computed in order to obtain y[n]. Globally, the calculations encompass two DFTs, one point-wise multiplication and one inverse DFT. The algorithm has a complexity of $O(L\log_{2}\left(L\right))$ if resorting to decimation-in-time Fast Fourier Transform (FFT) algorithms, as opposed to classical direct computation of DFTs, or methods such as the Goertzel algorithm ($O(L^{2})$) [22].

Let max{y[n]} and $\bar{y}$ denote the maximum and absolute average values of y[n], respectively. The detectability criterion consists of comparing the ratio $r:=\max\left\{y\left[n\right]\right\}/\bar{y}$ with a user-defined threshold *T*. If r>T, then a detection has been made.

Nevertheless, after the first successful detection, the signal may be completely or just partially inside the block. Considering the latter scenario, the determination of Times Of Arrival (TOA) could produce defective results. Such uncertainty suggests that the first detection corresponds only to an intermediate step of the overall decision process since only after a second detection in the consecutive sampling cycle is it possible to guarantee that the complete acoustic signal is contained within the FIFO buffer. It is only at this point that the matched filter can be run over data obtained in two consecutive sampling cycles, to obtain the exact TOA, which corresponds to max{y[n]}, of the incoming signal at each hydrophone.

**Figure 5:**
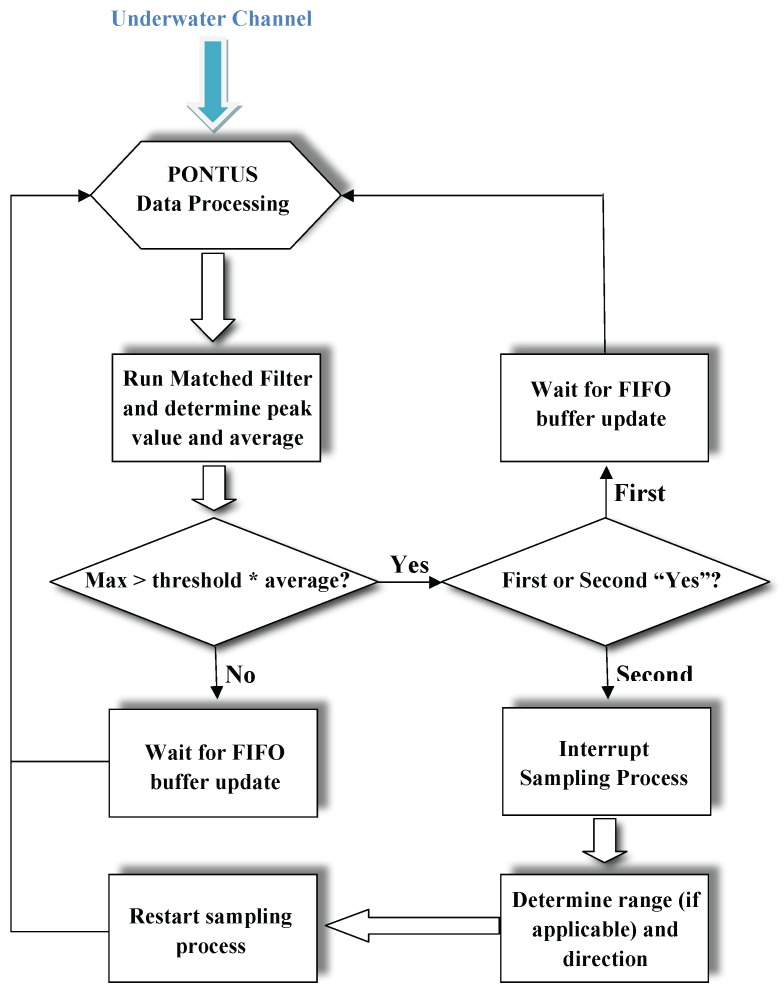
Detection scheme flowchart.

In short, the overall detection scheme follows the flowchart depicted in Figure 5.

Finally, the TDOA measurements are derived from the TOAs concerning all hydrophones, as explained in the sequel.

The implemented 127-chip Gold code allows for 129 simultaneous users, each one associated with a unique orthogonal code. In practice, this corresponds to a mission setup resembling that of a GPS localization system, featuring a constellation of 128 transponders and one (PONTUS) master receiver (more receivers can operate in the field as long as they do not interrogate the transponder network). Hence, assuming there are *N* transponders, the software routines associated with the detection scheme flowchart depicted in Figure 5 would have to be run *N* times, and *N* different matched filters would have to be stored in memory. Particularly for this work, each matched filter consists of 1270 floats, i.e., 3810 bytes, which means 128 matched filters would represent 635 kilobytes, which poses no challenge whatsoever to the hardware embedded in PONTUS. In terms of computation time, the routine described in the aforementioned flowchart yields an average running time of 4 ms. Thus, considering 128 transponders, the total time of computations would amount roughly to 512 ms, which is approximately half of the default 1-s sampling time. Therefore, even when accounting for extra computations and additional routines besides the one depicted in the flowchart, the system is capable of handling 128 receivers.

### 3.4. Data Processing

Sound waves generated by acoustic projectors propagate in water in a spherical pattern, and their curvature impacts on the TDOAs. However, given the length of the array baselines and the distance between the emitter and the hydrophones, the arriving wave can, in general, be approximated by a Planar Wave (PW).

The problem of localization in a reference coordinate frame based on PW and Spherical Interpolation (SI) methods was studied in [23], where the authors concluded the PW technique to be more effective and less sensitive to sensor noise when compared to the SI. This conclusion holds as long as the ratio between the baseline and the slant-range of the transponder is greater than 4%.

**Figure 6:**
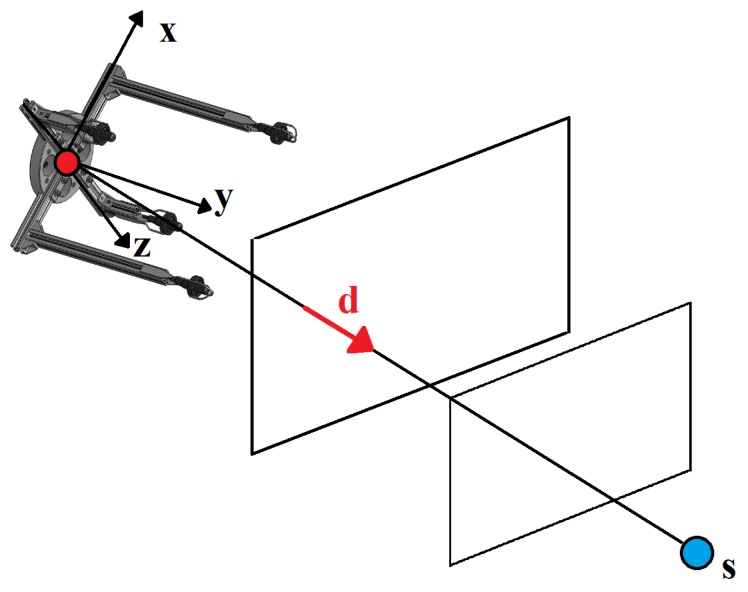
Planar wave approximation.

The problem setup, illustrated in Figure 6, can now be introduced. Consider two frames, an inertial frame denoted by {I} and a body-fixed frame denoted by {B}. The origin of {B} corresponds to the centroid of the array, and the relation between both frames can be expressed by means of a translation and a rotation matrix, RBI∈SO(3), denoting a rotation from {B} to {I}. Let $d\in R^{3}$ be a unit vector that represents the direction of the source expressed in {B}. Suppose that there are *N* receivers and that their position with respect to {B} is pnB, where n∈N:={1,2,⋯,N}. The TDOA measurements between a pair of receivers according to the PW approximation are thus given by:

(2) δ[i,j]=ti−tj=−1cdTpiB−pjBi,j∈N,i≠j,

where $t_{n}$ is the TOA at receiver *n* and *c* is the speed of sound in water. Let *k* be the number of the total two-combinations for *N* receivers, given by k=N(N−1)/2. Gathering all possible TDOA combinations between pairs of receivers into a single vector gives:

(3) $$\Delta ={\left[\delta_{\left[1,2\right]}\;\delta_{\left[1,3\right]}\cdots \delta_{\left[N-1,N\right]}\right]}^{T}\in R^{k}.$$

The direction vector can then be written as the solution of a least squares problem, resulting in:

(4) $$d=-cS^{+}\Delta ,$$

where $S^{+}={\left(S^{T}S\right)}^{-1}S^{T}$ is the pseudo-inverse of S, with:

(5) S=p1B−p2BTp1B−p3BT⋮pN−1B−pNBT∈Rk×3.

Monte Carlo simulations were carried out in order to assess the sensitivity of the proposed sensor’s array given different DOA under the PW approximation. Specifically, a spacial scanning with a horizontal and vertical aperture of 180 degrees was implemented, considering a step angle of three degrees, totaling 3600 analysis points. For each point, 1000 randomized trials were run, and the corresponding azimuth and elevation angles were calculated from Equation (4). In each run, TDOA measurements were corrupted by additive white Gaussian noise with zero-mean and standard deviation 0.01/c s. The speed of sound in water was set to 1500 m/s. Regarding the nature of TDOA measurements, the added noise expresses small deviations in the range induced by the sampling frequency of the Analog-to-Digital Converters (ADCs) set to 250 kHz and by a misplacing of the receivers. Hence, according to Equation (2), the term 0.01/c stands for an average deviation (along the direction of arrival) between two channels of 1 cm, which, given the maximum length between two channels (30 cm), corresponds to an error of 3.33%.

**Figure 7:**
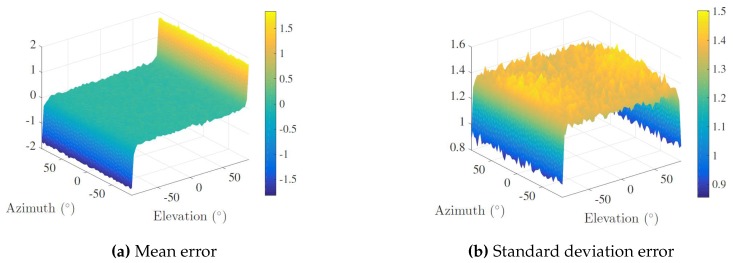
Elevation error analysis.

**Figure 8:**
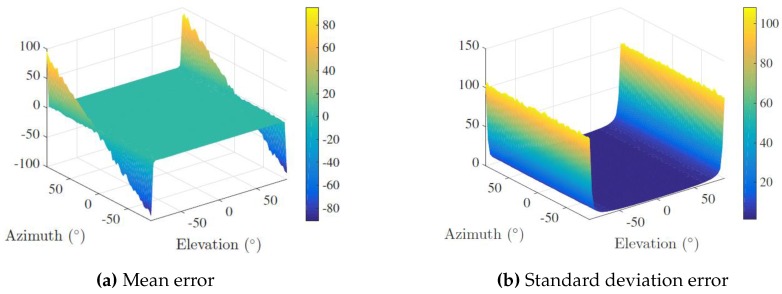
Azimuth error analysis.

The mean error and the standard deviation error of both angles was determined and averaged over the 1000 runs. The results are presented in Figure 7 and Figure 8 for elevation and azimuth, respectively.

Showing a good and consistent overall performance for most DOA, an immediate conclusion is that the array presents a greater sensitivity to noise in quadrant transition, i.e., at the ends of both horizontal and vertical apertures, although the determination of the azimuth comprises larger errors when the elevation is close to ±90 degrees. The physical explanation is intuitive: if the DOA is approximately collinear with the array’s *z* axis, TDOA measurements concerning the xy plane will be merely a product of ambient noise. This means that regardless of where PONTUS is facing and accounting for the planar wave approximation, it is not possible to disambiguate the azimuth of the target, i.e., all its admissible values (−180 to 180 degrees) are valid. This reflects a (well-known) singularity associated with the geometry of the problem.

Furthermore, resorting to the PW approximation and taking into account that the origin of {B} coincides with the centroid of the array, the range from the source to the origin of {B} can be approximated by averaging the range (ρ∈R) estimates from all receivers. The estimate for receiver *n* is computed from $\rho_{n}=ct_{n}$, and thus, averaging for all *N* receivers yields:

(6) $$\rho =\frac{c}{N}\sum_{n=1}^{N}t_{n}.$$

Finally, the position of the source relative to {B}, $s\in R^{3}$, expressed in {I}, is given by:

(7) sI=RBIdρ.

Note that the unfiltered complete estimate Equation (7) is directly achievable only in **O1** and **O3** operation modes.

### 3.5. System Calibration

Typical underwater missions are often concerned about georeferenced measurements in an inertial coordinate system, as given by Equation (7). The origin of this frame is usually located at a fixed known position at the surface, and all system measurements must be taken with respect to it. At this stage, unknown variables need to be accounted for, such as the speed of sound in water or an installation misalignment of the inertial unit due to a faulty assembly in the tube.

The calibration of PONTUS requires two sets of synchronized measurements. For instance, with regards to the selection of a ground-truth reference, GPS data with Real-Time Kinematic (RTK) corrections is an accurate option for localization. Therefore, USBL measurements, with respect to the inertial frame, should match the above GPS reference, the latter also expressed in the inertial frame.

Hence, the two sets of *n* collected measurements and the calibration parameters that relate them can be expressed as follows:

(8) $$X_{GPS}^{T}=\alpha MX_{PONTUS}^{T}+t1^{T},$$

where $X_{GPS}$, $X_{PONTUS}\in R^{n\times 3}$ are matrices holding the stacked measurements (*x*, *y*, *z* coordinates) from the GPS and PONTUS systems, respectively, both expressed in the PONTUS INS coordinate frame. Regarding the former, the measurements correspond to the raw computed 3D inertial positions yielded by the INS-aided USBL acoustic positioning system. In turn, α∈R is a scaling factor that accounts for offsets on sound velocity propagation in water and DSP clock frequency (as the TDOA is measured resorting to the DSP clock); M∈SO(3) is a rotation matrix that compensates for any installation misalignment between the axes of the INS body frame and the axes of the USBL array frame; $t\in R^{3}$ is an offset vector that eliminates the misplacement of the GPS antennas, which, for obvious reasons, cannot be immersed; finally, $1\in R^{n}$ is an auxiliary vector whose entries are all one.

The implemented calibration technique stems from the Extended Orthogonal Procrustes (EOP) analysis [24], whereby a least squares minimization problem is solved in order to find the best estimates $\hat{\alpha }$, $\hat{M}$ and $\hat{t}$ that match the cloud of points in $X_{PONTUS}$ onto the cloud in $X_{GPS}$ as close as possible. In summary, the calibration algorithm is as follows: (1) place the USBL receiver at a fixed known position and equip the target with GPS (preferably aided by RTK corrections); (2) move the target along rich trajectories and collect measurements from both the GPS and USBL systems; (3) run the EOP method to find estimates of the three calibration parameters; (4) validate EOP results: if errors are above a certain threshold, update new parameters and jump to (2).

Experimental results of the calibration procedures are presented in Section 6.2.

## 4. System Hardware Ensemble

**Figure 9:**
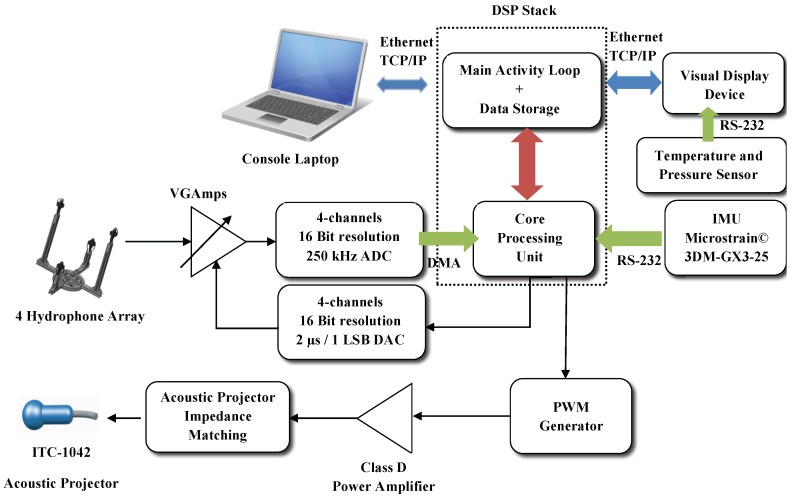
System diagram of PONTUS.

This section scrutinizes the blocks from Figure 9, as well as their relations. Overall, the electric characteristics of the system are presented, and an analysis of the major hardware components is made. For a better understanding, signal acquisition and signal generation stages are detailed in separate subsections. It is of considerable importance to stress that the hardware components ultimately chosen for our prototype were motivated by price-quality relationships and by their appropriate dimensions.

### 4.1. Power Supplies

The main power supply is a 236.8-Wh Lithium-Polymer (LiPo) rechargeable battery pack, assembled from four 16 Ah cells with 14.8 V nominal voltage each. A BATtery MONITor (BATMONIT) board, developed in-house, monitors the charge and discharge rates across the battery terminals and also the current, therefore preventing short-circuits, overloads or full discharges that may cause irreversible damages to the batteries. Moreover, the BATMONIT reads the output of an electrical reed switch bonded to the internal wall of the acrylic tube, providing an on/off toggle switch for PONTUS. This board monitors uninterruptedly the system with a residual power consumption (shutdown operating current <1μA) when compared to the overall current consumption of the system (>1.5 A).

The batteries can be externally charged or, in the case that PONTUS is mounted on a vehicle, power can be supplied by the vehicle. Since most electronic components require a fixed DC voltage level, in order to avoid voltage drops during operation, a galvanic isolated power converter is installed between the battery pack, or the external power supply, and the electronics.

The Digital Signal Processor (DSP) stack illustrated in Figure 9 requires both digital and analog power supplies. This supply duo is provided by two different high-performance small Traco© Power DC-DC converters in an isolated circuit. Similarly, the Voltage Gain Amplifiers (VGAmps) indicated in Figure 9 also need a bipolar power supply, provided by a Switch Low Noise Power (SLNP) board developed in-house. The SLNP power supply can range between 2.7 V and 24 V, allowing for a direct connection to the battery pack.

The visual display device that runs the GUI and the remaining digital circuits are all powered up by a third Traco© Power DC-DC converter. Finally, the Class D Power Amplifier depicted in Figure 9 is part of a signal amplification stage that is directly powered up by the main power supply.

### 4.2. Phase with Signal Conditioning Stage

Before being digitally processed, the raw acoustic signals (one for each hydrophone) are first passed through a bandpass second order Butterworth filter centered at 25 kHz. The resulting filtered signals are then passed through the VGAmps, whose gains are controlled via the digital to analog outputs of the DSP by resorting either to their energy or their instant maximum values as the adjustment criterion.

**Figure 10:**
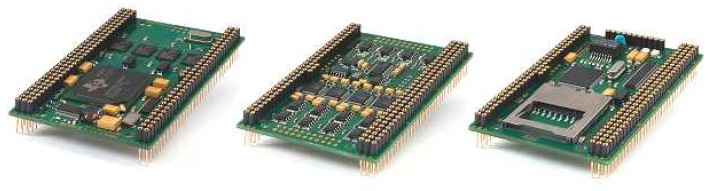
Core processing unit. From left to right: D.Module.C6713, D.Module.ADDA16, D.Module.91C111.

Recalling the diagram in Figure 9, the functionalities of the DSP stack are: core processing; hydrophone data acquisition; data and code storage. The stack consists of three boards, from the German manufacturer D.SignT, which are portrayed in Figure 10. From all of the commercially available DSPs, the one from D.SignT proved to be the most convenient, not only offering a flexible integration with other peripherals, namely data acquisition and TCP/IP network boards, but also excelling in its dimensions and specifications.

The main board is a high performance 32-bit floating-point D.Module.C6713 that operates at 300 MHz, featuring a Direct Memory Access (DMA) controller, which allows background data transfers to occur simultaneously with high-priority computations. This board is coupled (through a self-stacking design) to the 16-bit D.Module.ADDA16 board, which comprises four 16-bit resolution 250-kilo samples per second ADCs, in addition to four 16-bit resolution Digital-to-Analog Converters (DACs). Each one of the four hydrophones is connected to one VGAmp, whose output feeds the four ADC terminals on the D.Module.ADDA16.

### 4.3. Circuits for Acoustic Signal Generation

In operation mode O1, PONTUS emits an acoustic signal in order to trigger a reply from the transponder. The robustness and advanced design techniques of the DSSS signals, previously discussed in Section 3.2, aim at improving the detection SNR. Moreover, the B-PSK modulation of the signals avoids abrupt changes at the terminals of the acoustic projector because phase changes are synchronized with the zero crossing of the voltage signal.

**Figure 11:**
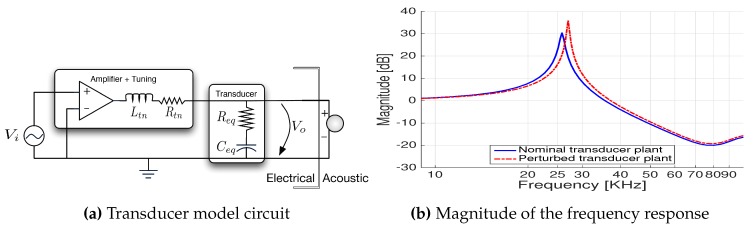
Impedance-matched transducer model.

To interface the acoustic projector, a switched power amplifier in addition to a step up voltage and impedance match circuit were required. For that matter, a new board, called PwrAmpD, was designed. In brief, the impedance matching circuit consists of a simple RLC circuit that uses a single solenoid (see Figure 11a), tuned to yield a very narrow band transfer function around the new 25-kHz resonant frequency, as seen from Figure 11b.

In summary, PwrAmpD is a highly efficient class D switch mode amplifier board in a full bridge configuration featuring an efficiency greater than 90% and low signal distortion. Moreover, it is optimized to drive acoustic projectors and to be driven by a Pulse Width-Modulated (PWM) waveform with a maximum frequency of 1 MHz.

The D.Module.C6713 integrates a complex programmable logic device that is used to implement a PWM modulator. The resulting PWM signal drives the PwrAmpD and, consequently, the underwater acoustic projector. Furthermore, using the impedance match circuit implemented in PwrAmpD, it is possible to fine-tune both the latter board and the acoustic projector in order to maximize the energy of the signal transmitted within the band of 20 to 30 kHz.

Nevertheless, amplification is just the first part of the signal emission optimization process with the objective of minimizing the percentage of failures at the reception side. To take advantage of the benefits of DSSS signals, the bandwidth limitations and non-linearities of the acoustic projector should be taken into consideration. Hence, taking full advantage of the works in [25], it was decided to implement the strategy depicted therein: a closed-loop design methodology for underwater acoustic projectors pulse-shaping. Very briefly, the bandwidth of spread spectrum signals does not lie completely within the bandwidth of the acoustic projector; therefore, the emitted signals will never fully resemble the desired ones. These sudden distortions at the transmission side will have negative consequences later when running the matched filters at the reception side. The idea exploited in [25] is that of modifying the transmitted signal in such a way that when passing through the acoustic projector, the dynamics of the latter shape the signal, making it resemble more the expected signal. This process of reverting the distortions is widely regarded as equalization, and its results applied to underwater acoustic projectors were successfully accomplished in [25].

## 5. Networks and Software Development

This section presents a short description of the software modules that compose PONTUS. It starts off with a brief look at the data networks across the hardware modules, finishing with the presentation of the GUI.

### 5.1. Communication Networks

PONTUS can communicate with external devices, for instance a console laptop, as suggested in Figure 9, and it can also receive external power and signals. Indeed, the SubConn© connector introduced in Section 2.2 provides to the outside:

(1) an Ethernet connection for communications between the processing core unit and a PC-based console laptop;
(2) a power supply terminal to charge the batteries without opening the tube;
(3) a single line for external synchronization with the pulse per second GPS generated signal, in mode (O3).

Owing to the DMA feature, while the FIFO buffers are being filled in the background, the main code thread is running matched filters on recent blocks of sampled data, further looking for events in the arriving signal. The subsequent computed direction and range are either stored or transferred to another device through the third card in the DSP stack. This card is an Ethernet Peripheral D.Module.91C111, providing a full-duplex 10/100-Mbit Ethernet interface. It allows data transfers between the DSP stack and the visual display device or a console laptop, whereby one can remotely configure, update or even communicate with PONTUS’ system without opening the tube.

Finally, there is yet another important set of measurements concerning the INS: the rotation matrix ℛBI. This matrix represents the attitude of PONTUS with respect to the inertial frame, typically the north-east-down frame. A high-performance, miniature MicroStrain© 3DM-GX3-25 (Williston, VT, USA) Inertial Measurement Unit (IMU) was chosen as the attitude and heading reference system.

### 5.2. Graphical User Interface

The GUI is an Android software-based application developed from scratch for this project.

Given the geometry and dimensions of the tube, the visual display device should be slim and small, in addition to being light and having low power consumption. An Android-based Samsung Galaxy S3 Model GT-I9305 (Suwon, Korea) was chosen as the hardware to run the GUI. Given all of its features and its interoperability and accounting for its storage capacity, the Galaxy S3 proves to be a cost-effective option.

**Figure 12:**
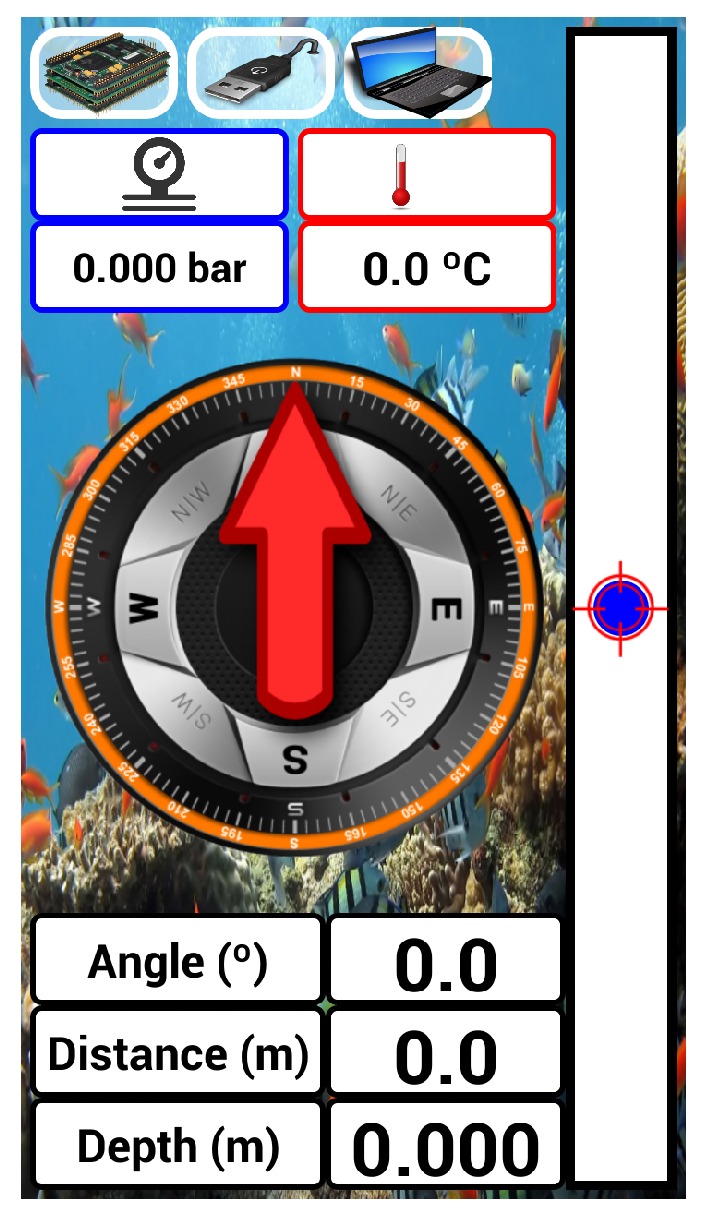
Graphical user interface of PONTUS.

The GUI shows the current outputs from the USBL, namely direction and range. It also provides temperature and pressure readings concerning the interior of the tube. Moreover, for the sake of debugging, the current state of inter-communications among devices is also displayed. A sketch of the resulting interface is depicted in Figure 12. A stylized compass and a vertical bar represent the bearing and elevation of the source expressed in {B}, respectively. Lastly, an alarm goes off if either the pressure or the temperature inside the tube exceed a safety threshold, 0.7 bar and $40^{\circ }$C, respectively.

The GUI was not tested underwater because experiments with scuba divers were not carried out. However, its performance was assessed and refined by using hardware-in-the-loop simulated measurements, namely range and directions. Furthermore, the GUI was an essential tool during the development stages, allowing one to check that a safe pressure and temperature were verified inside PONTUS prior to its deployment.

The first step in developing the GUI was to establish an Ethernet communication between the Android device and the DSP stack. This was only possible after replacing the native Android operating system with the Android open source distribution released by CyanogenMod© (open-source community).

**Figure 13:**
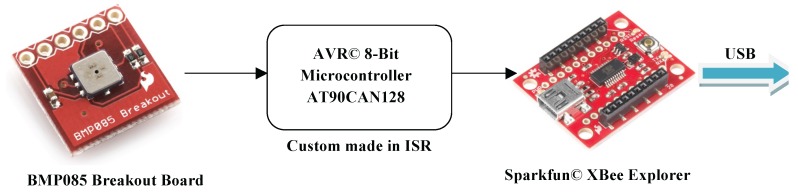
XBee diagram.

Besides Ethernet-based communications, the Android device reads the temperature and the pressure through a serial port interface. These readings are provided by a low-cost digital Bosch Barometric Pressure Sensor BMP085, which excels in its small size. The interface with the BMP085 is ensured by a board comprising an Atmel® AVR 8-Bit Microcontroller AT90CAN128 (San Jose, CA, USA), as depicted in Figure 13. This board is then connected to a Sparkfun© XBee Explorer USB (Niwot, CO, USA) so as to enable USB connections. Hence, the Ethernet interface of the DSP stack had to be redefined as an USB peripheral resorting to a USB/Ethernet adapter. Since the latter works only if connected to a host, the Android device must act as a host. Therefore, USB peripherals can be attached to (and powered up by) the Android device simply by using an On-the-Go (OTG) cable.

**Figure 14:**
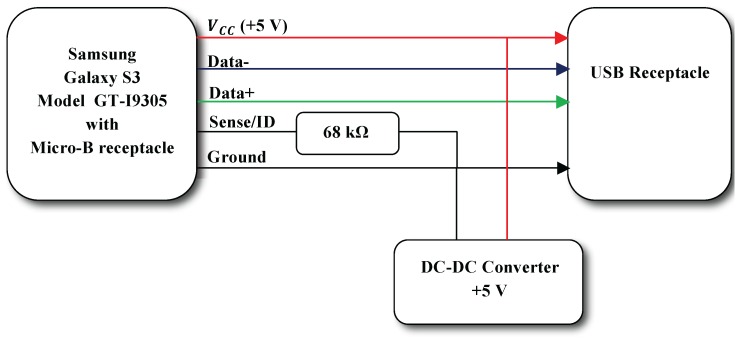
OTG cable with charging capabilities.

Consequently, the Android device, whose input data port is based on the micro-B connector, will have its battery charged through the OTG cable. Unfortunately, OTG cables are fabricated as data receivers, thus disabling power charging capabilities. In order to tackle this design issue, a small change is implemented in the OTG cable, as explained by the diagram in Figure 14. The resistance value can be found by testing the range of possible values concerning the USB standard.

With the Android device now available through an Ethernet port, communications with the DSP stack and/or with an external console are attainable through an Ethernet switch.

**Figure 15:**
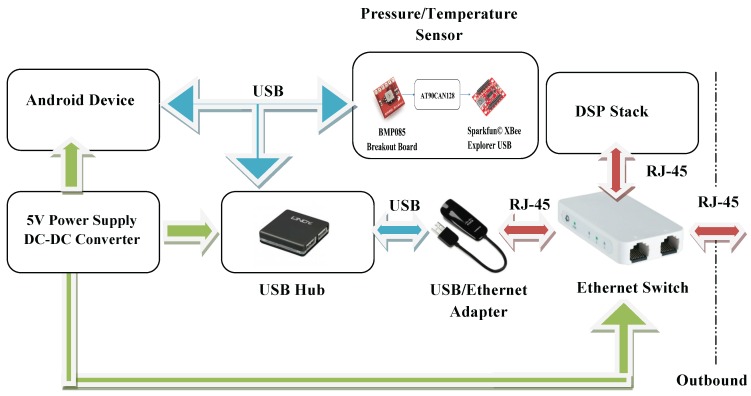
Visual display device and its dependencies. Green arrows represent single power lines.

In summary, the USB hub provides power to the Sparkfun© XBee Explorer USB (Niwot, CO, USA) and to the USB/Ethernet adapter, whereas a DC-DC converter powers the Android device and the Ethernet switch. In turn, the Android device powers the USB hub. The detailed description of the power and data transfers is enclosed in Figure 15.

## 6. Experimental Results

**Figure 16:**
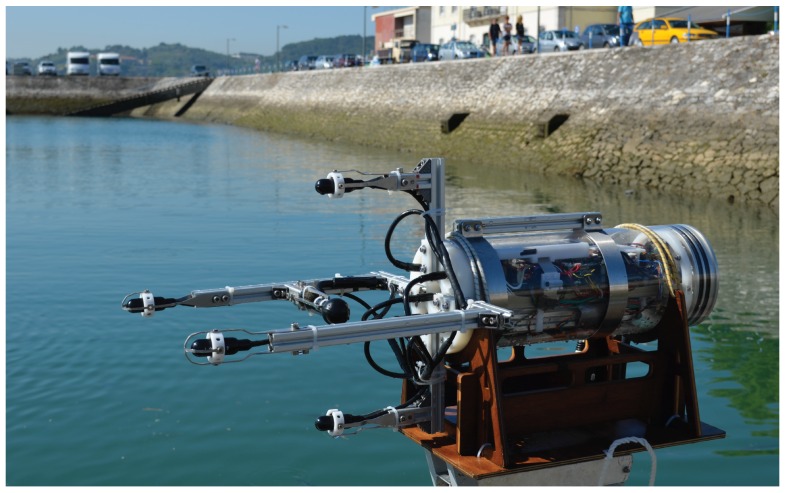
PONTUS prototype.

This section presents two sets of static and dynamic experimental results under mode of operation O1, wherein PONTUS and an acoustic transponder were placed underwater and range and bearings were collected during the experiments. It is important to stress that the aim of the static operation was to assess the repeatability (test-retest reliability) of the USBL system. In other words, the precision of the system was analyzed given different distances between PONTUS and a target, both placed at the bottom of a lake. In turn, the dynamic test consisted of a long-term operation for the evaluation of the overall USBL system in the presence of time-varying physical quantities, under harsh conditions imposed by the environment. The final prototype can be seen in Figure 16.

**Figure 17:**
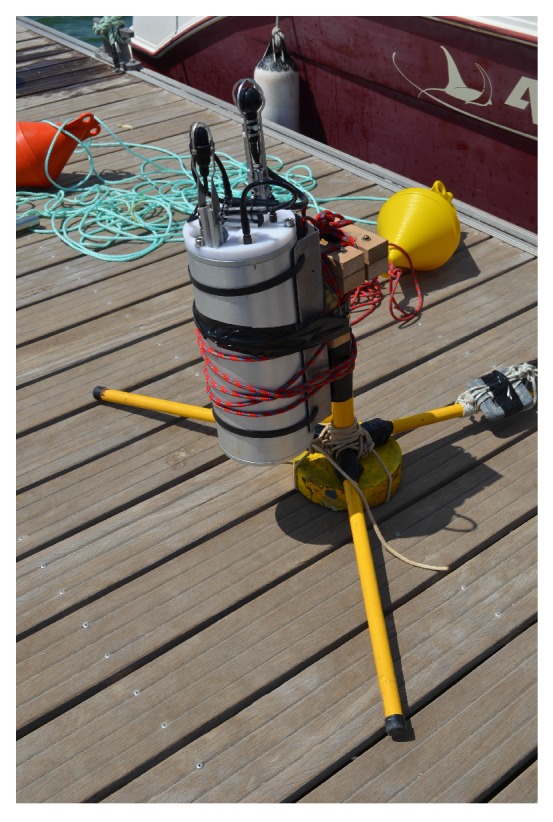
Acoustic Target (AT): the by-product of PONTUS.

The experiments reported herein rely on a transponder system that can be described as a subset of the USBL system prototype. The transponder listens for ping requests sent by PONTUS and replies to them with a predefined signal after a predefined delay. For this purpose, the transponder only needs a receiving channel, therefore one hydrophone, and does not need the IMU nor a visual display interface. In summary, the transponder system inherits from the previously described system the following blocks: the DSP stack with the three modules depicted in Figure 10, one AGCamplifier board designed at ISR, the emission power amplifier board PwrAmpD, a battery and a bank of DC converters and, finally, one acoustic projector and one hydrophone acting as the emitter and the receiver, respectively. Additional electronics were also added for coupling the transmitting and receiving circuits to the same acoustic projector to avoid high transmission voltages across the receiving AD converters when replying to the ping requests. For the sake of comprehension, the by-product will henceforward be designated as the Acoustic Target (AT). Figure 17 shows the AT (gray cylindrical shape) right before diving.

### 6.1. Static Operation

The position of PONTUS changed between trials, whereas the AT remained at the same location throughout the experiments. Due to the proximity of the apparatus to the seabed, multipath was expected to corrupt every set of measurements. Consequently, secondary trajectories associated with multipath could be wrongly interpreted as valid detections. This problem of ambiguity in the implementation of detection schemes is an active field of research, with many contributions available in the literature; in particular, the reader is referred to [26,27], which present new algorithms for underwater positioning based on an LBL configuration. Nonetheless, in order to overcome the problem of multipath propagation, real-time data classification algorithms able to detect invalid measurements were implemented. Specifically, we resorted to the outlier removal algorithm presented in [28], where its performance was successfully validated within the scope of USBL acoustic positioning systems.

**Figure 18:**
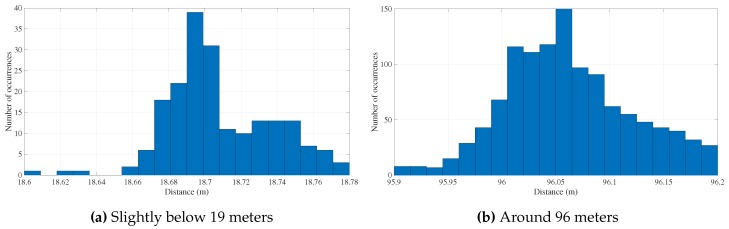
Histogram of two stationary tests.

Hence, the results shown below have already been through a stage of outlier removal, whereby all data considered to be corrupted were rejected. First, we compare the two histograms depicted in Figure 18. Since both acoustic elements were placed at unknown fixed positions at the bottom, the comparison between histograms illustrates the system performance in two distinct situations.

The set of points in Figure 18a is dispersed within a range of 20 cm, whereas the set depicted in Figure 18b occupies a larger interval, around 30 cm. Moreover, in Figure 18b, the central value does not stand out as evidently as in Figure 18a, i.e., it presents slightly large variation from the mean, thus a larger standard deviation. These two observations combined suggest a deterioration in the precision of the system with increasing distance, which is somehow expected when accounting for signal to noise degradation and for multipath interference that was not removed by the filter.

Overall, Table 1 incorporates a series of seven static tests, where range measurements were collected and the mean and standard deviation were subsequently determined. For instance, tests Number 2 and 4 reveal the biggest standard deviations among the other tests, implying that either the number of points was not enough to reduce the data dispersion or else the tests themselves were greatly affected by multipath.

The difference in the number of test points is justified by a coherence in terms of running time, that is each test lasted approximately the same time. Moreover, each one corresponds to a different location, therefore to a different geometric configuration. As shown in Table 1, the shorter the distances are, the more invalid measurements (outliers), thus less (valid) points, were collected (notice that each test corresponds to 1200 cycles). Accordingly, a brief analysis of the standard deviations indicated in Table 1 allows us to conclude that, in particular for short ranges (<100 m), the multipath is indeed a more important factor than the increasing distance, since the number of invalid points carried is more significant than the standard deviations. If any conclusion can be attained, it is that outliers are more frequent when the distance shortens, not only because the planar wave approximation weakens, but also because reflections from the bottom grow stronger. Still, regarding only these valid measurements, PONTUS shows a consistent performance despite the caveat of working in a shallow environment.

**Figure 19:**
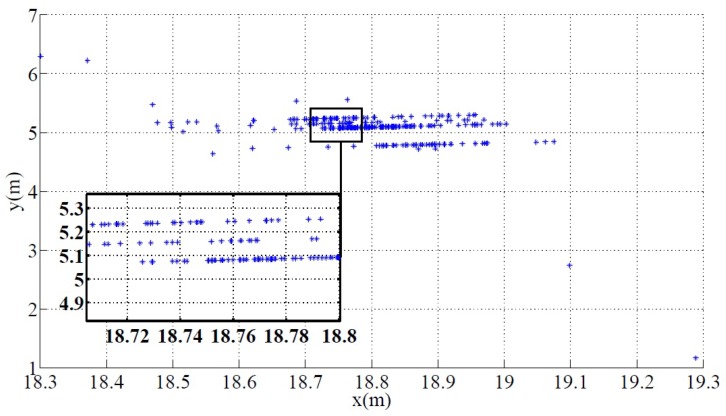
Position of the AT according to the body-fixed frame of PONTUS (Test No. 4).

Since range measurements are not enough to evaluate the performance of the USBL sensor, it is also fundamental to observe the results concerning direction readings. In fact, these readings are a better indicator of the presence of multipath in the underwater channel and also of its behavior. Observe now Figure 19, which corresponds to Test No. 4, where an XY scatter is represented to illustrate, in two dimensions, the relative position of the AT with respect to the body frame of PONTUS, {B}, considering the PW approximation method. First of all, if one were to approximate the scatter points with straight lines, clearly, there are three standing regions of greater density. This fact is not surprising, and each hop between two different contiguous regions is primarily related to the resolution of the system in terms of range determination. Recall the 250-kHz sampling frequency, which when associated with the speed of sound in water yields a resolution of approximately 6 mm. This notwithstanding, these quasi-straight lines are the result of a consistent multipath scenario and can be explained by the bottom reflection. Nonetheless, in terms of directions, the system presents a resolution close to the theoretical limit of resolution.

Furthermore, it is obvious that there are still some outliers depicted in Figure 19, even after all corrupted data have been rejected. This means that detectability algorithms (which are not flawless, i.e., not able to filter some false positives) might need an adjustment in order to improve the multipath clustering. Indeed, the direct path does not always imply greater energy, which means indirect/secondary trajectories are being wrongfully determined as if resulting from a direct path. Thus, even when accounting for the harsh conditions imposed by the environment, these results show that PONTUS offers a reliable target tracking solution.

### 6.2. Dynamic Operation

The dynamic experimental setup consisted of PONTUS placed at a fixed position close to the surface while the AT was attached to a moving kayak. Convenience in observing and acquiring data in real time motivated our setup. The position in real time of the acoustic duo was provided by two GPS antennas rigidly linked through two aluminum bars. Since the centroid of the antennas did not coincide with the centroid of the acoustic systems, an offset will have to be estimated during the calibration phase, in addition to a factor and a rotation matrix, as previously introduced in Section 3.5.

Both PONTUS and the AT were approximately 1.5 m under the surface (with close to zero relative depth). The experiments were carried out in a lake of very shallow waters (maximum depth <5 m) with mild currents. The kayak described rich trajectories at mostly constant low speed (approximately 1 m/s). Moreover, since the relative depth was practically zero, most results presented in the sequel concern only the XY plane. It is important to stress that despite the fact that PONTUS did not move, this empirical decision does not affect in any way the obtained experimental results.

Within the scope of underwater missions, a sound velocity profile is usually obtained prior to the experiments. For this particular set of trials, a plausible value is initially assumed, specifically 1500 m/s, whose offset is then corrected during the calibration phase. This notwithstanding, taking advantage of the fact that one has access to the physical variables of the system, one can resort, for instance, to a filtering technique where the speed of propagation of the acoustic waves is explicitly estimated [29].

**Figure 20:**
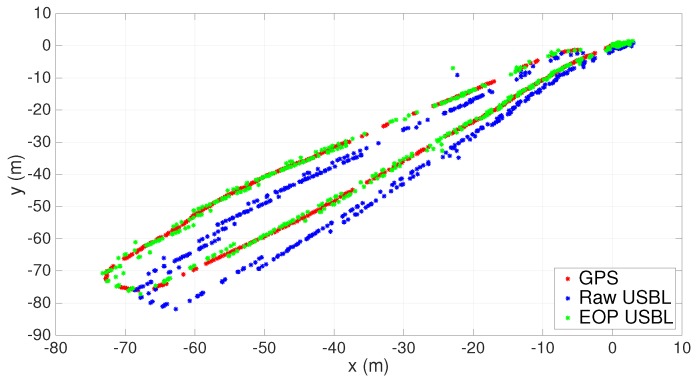
XY scatter before and after the Extended Orthogonal Procrustes (EOP)-based calibration. n=450.

**Figure 21:**
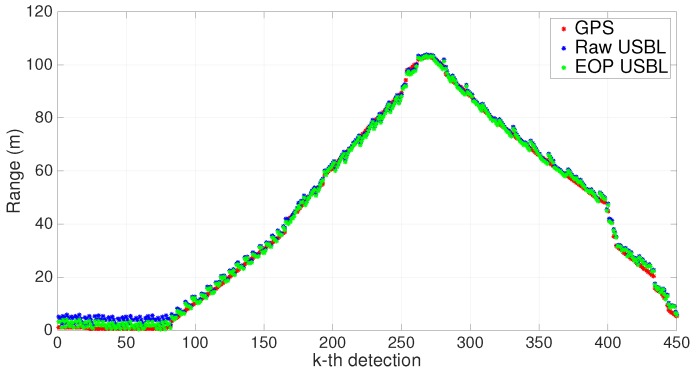
Range measurements before and after the EOP-based calibration. n=450.

In the following, the number of valid collected measurements is indicated by *n*. Results before and after calibration through the EOP analysis (see Section 3.5) can be seen in Figure 20 and Figure 21. An obvious aspect that stands out in Figure 20 is a correction of the installation rotation matrix due, almost certainly, to a faulty installation of the inertial unit. The corresponding bearing variation was estimated to be 3.5 degrees, which, when associated with increasing ranges, is responsible for greater data discrepancies following the aftermath of georeferencing. The scaling factor estimate, $\hat{\alpha }$, was 0.9985; therefore, the adjustment observed in Figure 21 is mainly the result of an offset correction.

**Figure 22:**
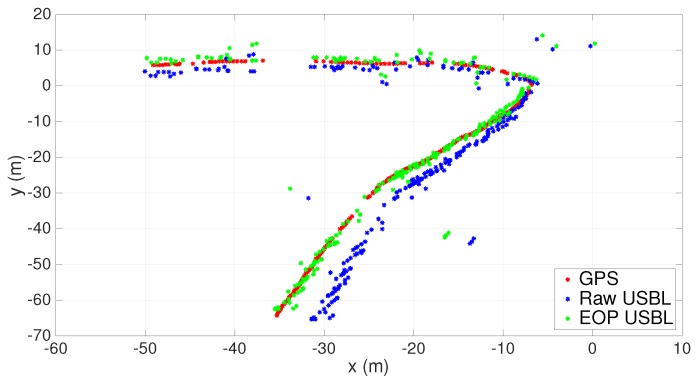
XY scatter using estimated calibration parameters. n=230.

**Figure 23:**
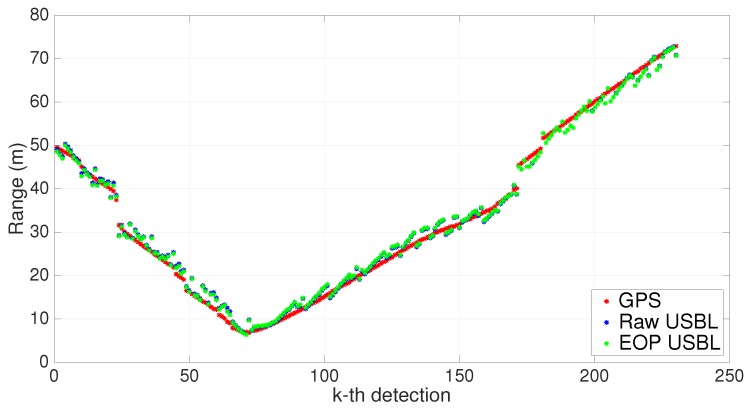
Range measurements using estimated calibration parameters. n=230.

In order to validate the EOP calibration process, one can use the estimated parameters resulting from the first trial and apply them to a new set of measurements collected during a second trial. The final results are depicted in Figure 22 and Figure 23. Once again, the correction in the rotation matrix stands out as the most important calibration parameter. Overall, using the previously estimated parameters in a second trial proved to be consistent with the new post-processed USBL measurements.

**Figure 24:**
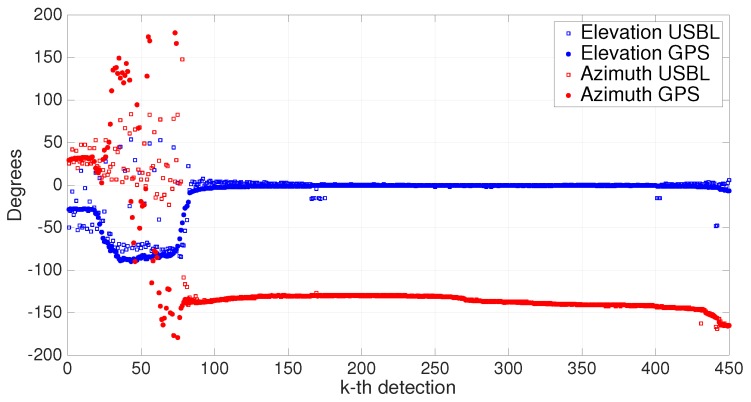
Comparison of elevation and azimuth angles.

To further assess the performance of PONTUS, Figure 24 shows a comparison between GPS directions and USBL directions. For the sake of comprehension, the directions were decomposed into elevation and azimuth angles. The corresponding range measurements are the ones depicted in Figure 21. As mentioned before, the relative depth was very close to zero, which means the elevation angle must be also very close to zero, as is shown in Figure 24.

It is clear that when the range is too close to zero, the USBL measurements are not reliable when compared to those of the GPS. This is due to the PW approximation, which, as noted in Section 3.4, degrades when the ratio between the baseline and the slant range of the target is less than for 4%. Since the baseline of the USBL array is 30 cm, if ranges are shorter than 7.5 m, then inaccurate direction measurements are expected. Hence, by discarding the values up to the 100th time index, a brief statistical analysis shows that the mean values of elevation and azimuth errors are $0.6787^{\circ }$ and $0.0221^{\circ }$, respectively. In turn, the standard deviations of elevation and azimuth errors are $4.7436^{\circ }$ and $1.4040^{\circ }$, respectively. Given the experimental setup conditions and the fact that the ranges in some cases exceeded 100 m, these are good results.

## 7. Conclusions

This paper presented the development of PONTUS, a prototype of an INS-aided USBL acoustic positioning system equipped with a GUI for diver-aided navigation. The motivation behind PONTUS arises from an absence of commercially available solutions that give access to the physical variables of the system, which, in turn, allow for the design of novel tightly-coupled algorithms for localization and navigation. Moreover, the paper presents a versatile and highly-configurable tool that can be used by divers or simply mounted on a vehicle. In particular, the geometrical configuration of the USBL array can be adapted to meet the requirements posed by the kind of mission that is performed. This notwithstanding, the implementation of DSSS signals opens the road for future applications consisting of complex underwater setups with multiple users, as well as for new distributed algorithms for relative and absolute target navigation and localization. Taking advantage of the access to the physical variables of the system, future work may also include the development and testing of novel Kalman filtering techniques to improve the outlier removal stage in both range and direction estimation.

A step-by-step description of the outer shell of the tool, as well as of its hardware ensemble and of its software, was detailed herein. A brief study on the signals involved in the operations was made, having been further addressed the techniques that allow their sampling and processing so that quantifiable measurements may return a 3D relative position of a target. In order to assess the repeatability of the USBL system, stationary experimental tests were conducted at several distances to verify the precision of the sensor, which, although under harsh conditions, was revealed to be consistent and in line with the expectations. Dynamic tests were also carried out that allowed one to perform a system calibration and further validate its operation, using ground truth data for performance evaluation.

## Figures and Tables

**Table 1:** Static range precision results.

Test No.	No. of Points	Mean	*σ*
1	93	11.403 m	56 mm
2	101	16.736 m	114 mm
3	197	18.708 m	29 mm
4	277	19.482 m	92 mm
5	216	19.677 m	38 mm
6	179	35.791 m	58 mm
7	1168	96.061 m	59 mm
